# A Hybrid System for Magnetic Hyperthermia and Drug Delivery: SPION Functionalized by Curcumin Conjugate

**DOI:** 10.3390/ma11122388

**Published:** 2018-11-27

**Authors:** Dorota Lachowicz, Agnieszka Kaczyńska, Roma Wirecka, Angelika Kmita, Wojciech Szczerba, Anna Bodzoń-Kułakowska, Marcin Sikora, Anna Karewicz, Szczepan Zapotoczny

**Affiliations:** 1 Academic Centre for Materials and Nanotechnology, AGH University of Science and Technology, al. A. Mickiewicza 30, 30-059 Krakow, Poland; akmita@agh.edu.pl (A.Kmi.); wojciech.szczerba@bam.de (W.S.); marcin.sikora@agh.edu.pl (M.S.); 2 Faculty of Chemistry, Jagiellonian University, Gronostajowa 2, 30-387 Krakow, Poland; agnieszka.kaczynska01@gmail.com (A.Kac.); karewicz@chemia.uj.edu.pl (A.Kar.); zapotocz@chemia.uj.edu.pl (S.Z.); 3 Faculty of Physics and Applied Computer Science, AGH University of Science and Technology, al. A. Mickiewicza 30, 30-059 Krakow, Poland; roma.wirecka@gmail.com; 4 Department of Biochemistry and Neurobiology, Faculty of Materials Science and Ceramics, AGH University of Science and Technology, al. A. Mickiewicza 30, 30-059 Krakow, Poland; anna.bodzon-kulakowska@agh.edu.pl

**Keywords:** magnetic nanoparticles, SPION, cationic chitosan, curcumin conjugate, magnetic hyperthermia

## Abstract

Cancer is among the leading causes of death worldwide, thus there is a constant demand for new solutions, which may increase the effectiveness of anti-cancer therapies. We have designed and successfully obtained a novel, bifunctional, hybrid system composed of colloidally stabilized superparamagnetic iron oxide nanoparticles (SPION) and curcumin containing water-soluble conjugate with potential application in anticancer hyperthermia and as nanocarriers of curcumin. The obtained nanoparticulate system was thoroughly studied in respect to the size, morphology, surface charge, magnetic properties as well as some biological functions. The results revealed that the obtained nanoparticles, ca. 50 nm in diameter, were the agglomerates of primary particles with the magnetic, iron oxide cores of ca. 13 nm, separated by a thin layer of the applied cationic derivative of chitosan. These agglomerates were further coated with a thin layer of the sodium alginate conjugate of curcumin and the presence of both polymers was confirmed using thermogravimetry. The system was also proven to be applicable in magnetic hyperthermia induced by the oscillating magnetic field. A high specific absorption rate (SAR) of 280 [W/g] was registered. The nanoparticles were shown to be effectively uptaken by model cells. They were found also to be nontoxic in the therapeutically relevant concentration in in vitro studies. The obtained results indicate the high application potential of the new hybrid system in combination of magnetic hyperthermia with delivery of curcumin active agent.

## 1. Introduction

Superparamagnetic iron oxide nanoparticles (SPION) are intensely studied due to their broad potential applications in medicine. Thanks to their superparamagnetic properties, SPION proved to be an excellent contrast for magnetic resonance imaging (MRI), one of the leading techniques in cancer diagnostics [1,2,3]. Recently, SPION have also gained attention due to their ability to induce magnetic hyperthermia (MHT) [4]. When an external alternating magnetic field of specific frequency is applied, it causes SPION to heat. Sustained temperatures above 42 °C destruct internal structures of the cells, leading to their death [5]. MHT allows for the local treatment of tumors with only moderate side effects, and may be considered well tolerated in comparison to other available therapies [6,7], because tumors are more susceptible to heat compared with normal tissue. Safety of the treatment depends also on the favorable biodistribution of the nanoparticles with their increased concentration in tumor tissue.

The magnetic properties of SPION are essential for their performance in MHT or as MRI contrast, thus they are important when SPION application in cancer therapy is being considered. Those can be tuned by the right choice of particles’ size and coating. To sustain the superparamagnetic behavior of iron oxide particles, the size of the single crystal should not exceed 20 nm [8]. For biomedical application purposes, SPION also need to be surface-modified by a well-chosen coating to increase their colloidal stability, biocompatibility, and to inhibit their elimination by the reticuloendothelial system (RES). Natural polysaccharides have often been used for this purpose, such as dextran [9], hyaluronic acid [10], alginate [11], and chitosan [12,13,14]. Recently, our group has proposed chitosan modified with trimethylammonium groups as a coating for SPION [15]. The resulting nanoparticles (SPION-CCh) were characterized by high colloidal stability due to the presence of an even surface charge. The obtained SPION with the average size slightly below 100 nm were the agglomerates of the small coated nanocrystals. Due to the presence of the polymer coating separating the nanocrystals, the cores preserved their superparamagnetic behavior, showing excellent magnetic properties. This coating was later successfully modified to attach monoclonal antibodies and used as an MRI agent [16].

Curcumin is a polyphenol of natural origin, which is extracted from the plant, Curcuma Longa L., and is known for its anti-oxidant and anti-inflammatory properties [17]. Numerous studies, both in vitro and preclinical, have also confirmed its potential as an anti-cancer agent [15,18]. Due to its hydrophobic character and low solubility in aqueous media, curcumin has been incorporated in various delivery systems to increase its bioavailability [19,20,21]. Polymeric conjugates have also been reported to effectively deliver curcumin [22,23]. Recently, a targeted anti-cancer was proposed, in which magnetic heating generated by gadolinium-doped nickel ferrite nanoparticles was used to trigger the release of curcumin from the thermosensitive polymeric matrix composed of amine-terminated poly-N-isopropylacrylamide and chitosan [24].

Here, we propose to combine the unique properties of SPION and curcumin to obtain a system with higher anti-cancer potential. Curcumin was first reacted with sodium alginate to obtain a water-soluble, negatively charged conjugate. This conjugate was then used to form a second, outer layer on SPION-CCh. The physicochemical and magnetic properties of the obtained system were then studied to verify its potential usefulness in anti-cancer therapy.

## 2. Materials and Methods 

### 2.1. Materials 

Chitosan (low molecular weight), curcumin (puriss. pa, ≥94%, (curcuminoid content), ≥80%)), alginic acid sodium salt from brown algae (low molecular weight), FeCl_3_·6H_2_O (puriss. pa, ≥ 99.0%), FeCl_2_·4H_2_O (puriss. pa, ≥99.0%), and sodium chloride ammonia solution (25%, POCH S.A., puriss. pa, ≥99.0%) were used as received (all from Sigma Aldrich, St. Louis, MO, USA).

Cationic modification of chitosan, *N*-[(2-hydroxy-3-trimethylammonium) propyl] chitosan chloride (CCh), was obtained and characterized in accordance with the procedures described in the previous work [15]; its modification degree with trimethylammonium groups was indicated to be 62.2%. Millipore-quality water was used during the experiments.

#### 2.1.1. Synthesis of Curcumin and Sodium Alginate Conjugate (AA-Cur)

AA-CUR conjugate was obtained via Steglich esterification in the presence of dicyclohexylcarbodiimide (DCC) and 4-NN-dimethylaminopyridine (DMAP) as catalysts. Sodium alginate, DCC, and DMAP were used in a weight ratio of 20:2.5:1. All reagents were dissolved in DMSO and stirred for 1 hour at 25 °C in the atmosphere of the neutral gas. After that time, curcumin solution in DMSO (c = 0.152 mM; DMAP:curcumin molar ratio 65:1) was added and the reaction mixture was stirred for another 6 hours at 60–65 °C in the atmosphere of the neutral gas. The resulting solution was cooled down, dialyzed first against DMSO and then against water, and lyophilized.

#### 2.1.2. Synthesis of Uncoated SPION and SPION Stabilized with Cationic Chitosan/AA-Cur Bilayer (SPION-CCh/AA-Cur)

The synthesis of SPION and SPION-CCh was performed in water by co-precipitation of iron (II) and iron (III), following the procedure developed and described earlier [15]. To obtain uncoated SPION, the synthesis was conducted without CCh; in the case of SPION-CCh—in the presence of CCh. To introduce the AA-Cur layer, 5 mL of SPION-CCh solution was purged with neutral gas (Ar) for 2 min in the ultrasound pulsonic bath (Polsonic, Warszawa, Poland) at 20 °C. In the next step, 10 mL of water solution of AA-Cur (c = 1 g/L in 0.1 M NaCl) was added dropwise (the sonication of the solution was continued throughout the procedure) and the mixture was again purged with neutral gas for 2 min, and the system was sealed. The reaction was carried out in the room temperature for 30 min. To purify the resulting SPION-CCh/AA-Cur, magnetic filtration was used. The particles caught in the column were rinsed with water (5 mL) and then the obtained suspension was filtered by cellulose syringe filters (0.40 μm). The content of iron in the samples was determined by the classical colorimetric method based on absorbance measurements (Evolution 220 UV-Vis Spectrophotometer, Thermo Fisher Scientific, Waltham, MA, USA) of the complex of Fe(II) with 1,10-phenanthroline [16].

### 2.2. Methods

#### 2.2.1. Transmission Electron Microscopy 

The morphology of the SPIONs were determined using a transmission electron microscope (TEM) Tecnai TF 20 X-TWIN (FEI, Hillsboro, OR, USA). Nanoparticles were deposited onto ultrathin carbon film on holey carbon. The mean particle size and the size distribution for the synthesized nanoparticles were obtained by fitting round circles around the lattice fringes of the nanoparticles and measuring their diameter. 

#### 2.2.2. Small Angle X-ray Scattering Measurements

SAXS (Small-angle X-ray scattering) measurements were made in a flow-through capillary with a Kratky-type instrument (SAXSess, Anton Paar product, Graz, Austria) at 294 ± 1 K. That instrument has a low sample-to-detector distance of 0.309 m, appropriate for studies of dispersions with low scattering intensities. The determined intensity values were converted to an absolute scale according to Orthaber et al. [25,26]. The scattering vector magnitude, q, depends on the wavelength, λ, of the radiation (λ = 0.154 nm) as q = (4πn/λ)sinθ. Deconvolution of the SAXS curves was made with the SAXS-Quant software. Samples analysed with SAXS were used as prepared. The software, SASfit [27], was used for curve fitting.

#### 2.2.3. Vibrating Scanning Magnetometry Measurements

Volume magnetic properties of the dispersions were probed using a Vibrating Sample Magnetometer (VSM) LakeShore type 7407 (Carson, CA, USA). Temperature dependence of magnetization have been measured under magnetic flux of 100 Gs from 80 K to 170 K after zero field cooling (ZFC) and field cooling (FC). Saturation magnetization, magnetic remanence, and coercive field was determined from hysteresis curves measured on frozen and liquid dispersions, at 80 K (LN) and 290 K (RT), respectively, under a magnetic field up to 15 kOe.

#### 2.2.4. X-ray Photoelectron Spectroscopy Measurements 

X-ray photoelectron spectroscopy (XPS) measurements were performed using a PHI 5000 Versa Probe II (ULVAC-PHI, Chigasaki, Japan) spectrometer equipped with an Al Kα radiation source (E = 1486.6 eV). The operating pressure in the analytical chamber was less than 3 × 10^−7^ Pa. High resolution spectra were recorded with the analyzer pass energy set to 49.95 eV. A dual-beam charge neutralizer was used to compensate the charge-up effect. All binding energies were corrected to C–C line at 284.8 eV. The spectrum background subtraction was done using the Shirley method. Data analysis software from PHI MultiPak was used to calculate elemental compositions from the peak areas.

#### 2.2.5. Magnetic Heating

The apparatus for measuring the magnetic hyperthermic effect consisted of a chamber in which 0.4 mL of the sample was placed in a polymer Eppendorf test-tube together with an optical fiber temperature sensor connected to the temperature measurement system. The sample was placed in the middle of the induction coil (using polymer falcon with a diameter equal to 2.5 cm, providing thermal isolation of the heated nanoparticle suspension. The magnetic field was generated by an induction heating generator (EASY Heat 0224 FFC CE, St. Louis, MO, USA). The induction coil was cooled down using a water cooling system (TEXA TCW12NBSBCP0000, Pegognaga, Italy). The measurements were performed varying the power of the generator in the range of 0.6–1.6 kW that corresponded to the magnetic field strength (H): 23.8–35.7 kA/m; H = 27.57 kA/m was used to determine SAR. The actual temperature of the sample was recorded in real time. The heating rate was determined as an initial (up to ca. 200 s) slope of temperature vs time plot that was fitted with linear regression [28].

#### 2.2.6. Other Methods

Fluorescence emission spectra were obtained using a Fluorescence Spectrometer LS55 Perkin Elmer precisely at 25 °C. The FTIR spectra were recorded in the range of 400 to 4000 using a Thermo Nicolet iS10 FTIR spectrometer (Thermo Scientific, Madison, WI, USA) with an ATR accessory. The samples for FTIR analysis were prepared by lyophilization. Dynamic light scattering (DLS) and zeta potential measurements were done using a Malvern Nano ZS apparatus (Malvern Instrument Ltd., Worcestershire, UK). Zeta potential was determined using Laser Doppler Velocimetry (LDV, Malvern Instrument Ltd., Worcestershire, UK). Thermal analysis was done by using a thermogravimetric analyzer (TG) Q600 (TA Instrunents, New Castle, DE, USA). Analyses were performed under an inert atmosphere. The gas flow rate was 50 mL/min, and the heating rate (β) was 20 °C/min.

Fluorescence imaging of cells was performed using a Nikon inverted microscope Ti-E (Nikon Instruments Inc., Melville, NY, USA) with a confocal system Nikon A1 with a 405 nm diode laser for excitation. The images were acquired in the emission mode with a Plan Apo 100×/1.4 Oil DIC lens (Nikon Instruments Inc., Melville, NY, USA). The emission images were collected using a 458 nm barrier filter to remove the fluorescence background originating from the cells. The resolution of the images was 2048 × 2048 pixels. Slides for confocal microscopy imaging of cells were prepared by fixing curcumin-loaded cells in 1% paraformaldehyde (PFA) in phosphate buffer (PBS) for 15 min at 4 °C.

#### 2.2.7. Biological Stud Studies

Fibroblasts cell (NIH3T3 line) were cultured in DMEM high glucose in a damped incubator (Memmert GmbH & Co. KG, Schwabach, Germany) at a temperature of 37 °C, 5% CO_2_. The media have been complemented of 10% FBS, streptomycin (100 U/mL), and penicillin (100 g/mL). The cells were subcultured for every 2 days until a suitable number of cells was received for the test. After the culture had reached around 80% confluence, the cells were trypsinized, seeded on sterile 96-well plates (2.5 × 10^4^ cells/cm^2^), and incubated for 24 h.

The cytotoxic activity of SPION-CCh and SPION-CCh/AA-Cur in fibroblast cell (NIH3T3) was estimated by the MTT (3-[4,5-dimethylthiazole-2-yl]-2,5-diphenyl tetrazolium bromide) dye conversion assay. Cells (4 × 10^4^) were cultured in 0.1 mL volume of culture medium in a 96-well plate in the presence of different concentrations of iron in obtained systems dissolved in the medium. After 24 h, the cells were washed once and further incubated for 1 h with MTT dye. The obtained blue formazan precipitate was dissolved using solubilization buffer (5 mM HCl in isopropanol) and kept for 2 h at 37 °C. The absorbance at 570 nm was measured using a microplate reader (Infinite M Nano, Tecan, Männedorf, Switzerland). Each result was presented as a mean of the three independent experiments, each of them performed in triplicate. SD was also calculated and presented for each mean value. The significance of the differences between cell viability values was determined with a Student’s t test for two-group comparisons. In all the cases, a probability value (p-value) of less than 0.05 was considered to be significant.

## 3. Results and Discussion

### 3.1. Morphology and Chemical Characterization of Obtained Systems 

**Figure 1:**
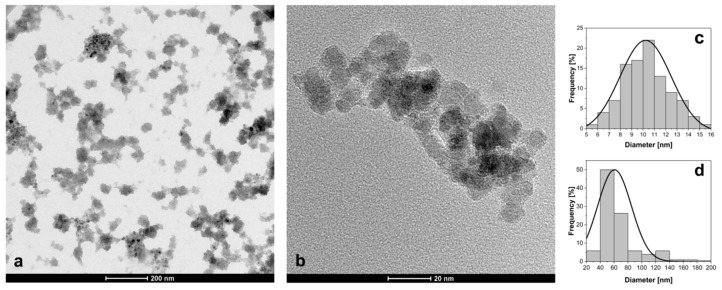
(**a**,**b**) TEM micrographs of SPION-CCh-AA-Cur; size distribution of (**c**) magnetic cores and (**d**) agglomerates.

Morphology and size of the obtained nanoparticle system (SPION-CCh/AA-Cur) was determined by transmission electron microscopy (TEM). The image of SPION-CCh/AA-Cur shows agglomerates formed by magnetic cores coated with a polymeric bilayer (Figure 1). The observed magneto-polymeric agglomerates had an average size of 69 ± 31 nm (Figure 1d) with cores well visible in the polymeric matrix. The sizes of the magnetic cores were found to be in the range of 5 to 16 nm, with an average diameter equal to 10.2 ± 2.1 nm (Figure 1c). 

**Figure 2:**
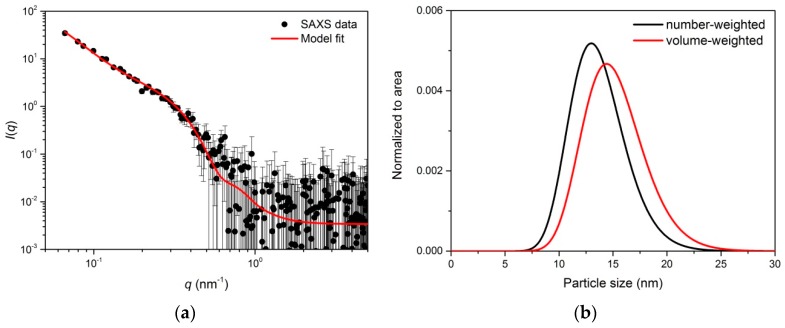
Scattering curve of SPION-CCh-AA-Cur with the fitted model involving two populations of spherical particles (cores and agglomerates) (**a**) and size distribution functions obtained from the model (normalized to the total number of particles) (**b**).

Small-angle X-ray scattering (SAXS) measurements of SPION-CCh/AA-Cur revealed a bi-log-normal size distribution for the iron oxide cores (Figure 2). The scattering curve was fitted with the mathematical model using an analytical form factor of a sphere and lognormal size distribution of the radii. The cores have a moderately broad size distribution. Their mean diameter is 13.7 ± 2.5 nm. A significant fraction of cores contributes to agglomerates. The agglomerate size modelled with a mass fractal with an exponential cut-off is approximately 44 nm. It is built of elementary particles with a size of 18.6 nm. The difference between the size of primary particles and the size of a core can be interpreted as the thickness of a stabilizer layer of the primary particles that cannot be distinguished by SAXS due to the low contrast between the stabilizer and the solvent. The fractal dimension of nearly three suggests that the agglomerates are close to dense packed globular structures. Their modelled size is approximately 44 nm, which is in agreement with the results of the TEM analysis (Figure 1d).

**Figure 3:**
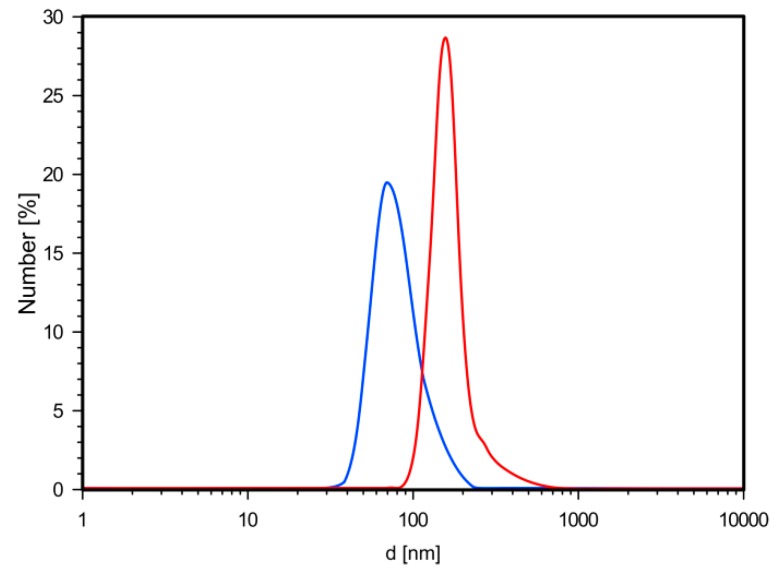
Distribution profiles of the hydrodynamic diameters obtained from DLS measurements for the SPION-CCh (red line), SPION-CCh/AA-CUR (blue line).

Based on dynamic light scattering measurements of SPION-CCh/AA-Cur, we have noticed that the coating procedure had a positive effect on the size and surface charge of the agglomerates. Coating of the SPION-CCh with the anionic conjugate, AA-Cur, changed the zeta potential of the nanoparticles from +34 to −35 mV (see Table 1). It can be seen that SPION-CCh/AA-Cur form smaller agglomerates, whose average size do not exceed 60 nm (Figure 3). A possible explanation may be that the strong polycation-polyanion interactions result in a more compact structure of the coating.

**Figure 4:**
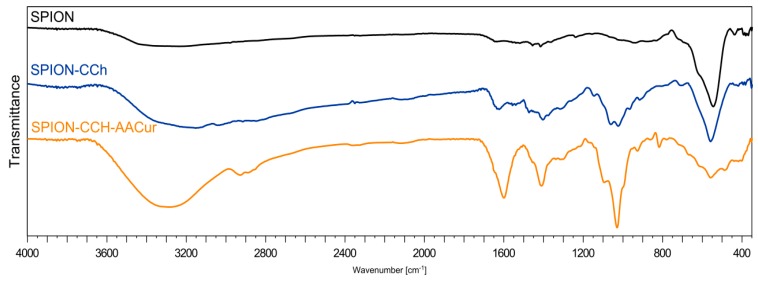
FTIR spectra of SPION without polymer, SPION-CCh, SPION-CCh-AA-CUR.

To confirm the presence of cationic chitosan(CCh) layers and alginic conjugate (AA-CUR) layers in the obtained systems, FTIR analysis was performed. Figure 4 shows the FTIR spectra of magnetic iron oxide nanoparticles without surface modification (SPION), stabilized with the cationic chitosan layer (SPION-CCh), and with the polymeric bilayer containing curcumin conjugate (SPION-CCh/AA-Cur). At the FTIR spectrum, SPION bands at 550 cm^−1^ and 625 cm^−1^ can be seen, originating from Fe–O stretching vibrations of the iron oxide cores [29]. The weak band at 1620 cm^−1^ and the broad band around 3350–3400 cm^−1^ can be attributed to the adsorbed water and surface hydroxyl groups (Fe–OH) [30]. The FTIR spectra of SPION-CCh and SPION-CCh/AA-Cur confirm the presence of a cationic derivative of chitosan. A broad peak in the range from 3500 to 2950 cm^–1^ can be interpreted as the overlap of amine (–NH_2_, –NH–) and hydroxyl (–OH) groups’ stretching vibrations characteristic for the chitosan derivative (CCh). The spectra also show a band at 1474 cm^−1^, characteristic for the methyl fragment of the trimethylammonium group; the band at 1541 cm^−1^, characteristic for the primary amine [31]; and a band at 1625 cm^−1^, characteristic for amide I [32,33]. 

The bands at 1021 cm^–1^ and 1056 cm^−1^ (characteristic for a C–O–C bond) could be observed in both systems with the polymer coating, while the band at 560 cm^−1^, which can be assigned to the Fe–O bond [34], is visible in all three systems studied. The spectrum obtained for SPION-CCh/AA-Cur exhibited also the bands characteristic for the conjugate: At 1600 cm^−1^ (asymmetric stretching vibration of COO groups) [35], at 1410 cm^−1^ (symmetric stretching vibration of COO groups), and at 1027 cm^−1^ (elongation of C–O groups) [36]. The results confirm that SPION-CCh were successfully coated with AA-Cur.

**Figure 5:**
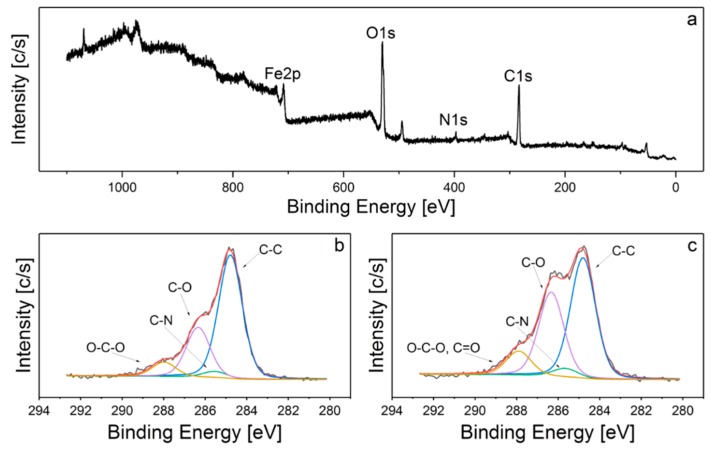
XPS spectra of: (**a**) SPION-CCh-AA-Cur survey, (**b**) SPION-CCh C1s, (**c**) SPION-CCh-AA-Cur C1s.

To further investigate the structure of SPION-CCh and SPION-CCh/AA-Cur systems, their surface chemical bonds were studied with the XPS wide scan. Figure 5a shows the survey spectrum of the SPION-CCh/AA-Cur. The peaks observed at 710, 530, 400, and 285 eV correspond to the Fe2p, O1s, N1s, and C1s lines, respectively. C1s spectra of SPION-CCh and SPION-CCh/AA-Cur were curve fitted with four different peaks with an equal full width at half-maximum (FWHM) using a Shirley-type background subtraction (Figure 5b,c). The binding energies of 284.8, 285.6, 286.3, and 287.9 eV can be attributed to the C–C/C–H, C–N, C–O, and O–C–O/C=O groups of the polysaccharide coating, respectively [37,38]. The atomic content ratio of C to O (C/O) in the SPION-CCh/AA-Cur is smaller than that in SPION-CCh, which implies a decrease of the relative content of CCh in the system. Moreover, the content of C=O and C–O groups in the SPION-CCh/AA-Cur is significantly higher than that in SPION-CCh. Additionally, the intensity of the iron peak (710 eV) slightly decreases in SPION-CCh-AA-Cur. These results confirm the presence of the AA-Cur layer in the SPION-CCh/AA-Cur system.

**Figure 6:**
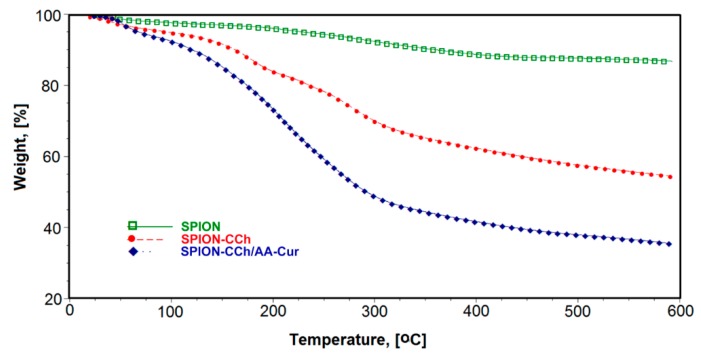
Thermogravimetric analysis of the SPION systems: Red—native SPIONs; green—SPION-CCh; blue—SPION-CCh/AA-Cur.

The TGA curves for SPION and surface modified SPION-CCh and SPION-CCh/AA-Cur are shown in Figure 6. For uncoated SPION, a small weight loss was observed below 200 °C, which could be attributed to the removal of residual water and another one visible between 200 °C and 400°C, which can be related to the removal of surface hydroxyls [39]. No weight loss in temperatures higher than 400 °C occurred. The total weight loss for SPION was calculated as 11.3%. For SPION-CCh and SPION-CCh-AA-Cur, a much higher weight loss was registered both below 200 °C and in the range of 200–400 °C. The first step can be assigned to the loss of residual water, as both layers (CCh and AA-Cur), consisting of hydrophilic polymers, bind in their structure significantly more water than uncoated SPION. Above 200 °C, decomposition of the polymeric layers starts. It is most pronounced in temperatures between 200 °C and 400 °C: Calculated weight loss in this range equaled 37.6% for SPION-CCh and 58.3% for SPION-CCh/AA-Cur. This can be easily explained by the difference in the relative weight percentage of the polymer to the inorganic core in both systems. Decomposition of the remaining polymer, most probably the CCh intercalated into the structure of iron oxide during the particles’ formation step, continues up to 600 °C. The obtained results confirm the presence of the CCh layer and appropriate bilayer on the surface of SPION-CCh and SPION-CCh/AA-Cur, respectively.

### 3.2. Magnetic Properties of SPION-CCh-AA-Cur

**Figure 7:**
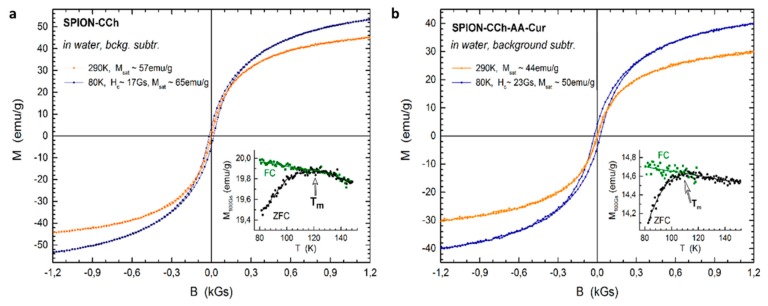
The low field region of magnetization loops of SPION-CCh (**a**) and SPION-CCh-AA-Cur (**b**) sample at selected temperatures (80 K and 290 K). Inserts present the temperature dependence of the ZFC and FC magnetization of SPION-CCh (**a**) and SPION-CCh/AA-Cur (**b**) samples measured at warming under 100 Gs. T_m_ denotes the temperature of the maximum of ZFC profile.

To analyze the influence of the coating on the magnetization of the nanoparticles, the SPION-CCh and SPION-CCh/AA-Cur magnetizations were determined. The registered curves can be found in Figure 7. Vibrating sample magnetometry confirmed the superparamagnetic properties of SPION-CCh and SPION-CCh/AA-Cur at room temperature. A lack of coercivity and remanence on the magnetization curves was observed in both systems at room temperature, which means that the interparticle coupling within the agglomerates is negligible and thus the agglomerates can be considered as an ensemble of superparamagnetic cores. 

More evidence of superparamagnetic behavior was obtained from the temperature dependence of low field magnetization after zero field cooling and field cooling. At low temperatures, thermal energy is not sufficient to overcome the anisotropy energy barrier of a single particle. Thus, the ZFC and FC magnetization profiles differ significantly. Upon temperature increase, the equilibrium between anisotropy energy and thermal fluctuation energy can be reached and the two magnetization profiles should coincide. It is denoted as the blocking temperature of a superparamagnetic particle. However, for a finite size distribution of an ensemble of particles, the blocking temperature is also distributed accordingly [40,41]. Thus, ZFC and FC profiles coincide only for the temperatures that are higher than the blocking temperature of the majority of the particles probed. Since the coincidence temperature of ZFC and FC profiles is approximately 120 K in the samples probed, we expect that the volume of blocked particles (cores) at room temperature is negligible. 

No signature of the Verwey transition was observed in the temperature dependent profiles, suggesting that the particle cores are composed of maghemite. The saturation magnetization (M_sat_) of the SPION-CCh equals to 57 emu/g, which is lower than that of bulk magnetite (92 emu/g) [42] and γ-Fe_2_O_3_ (73.5 emu/g) [43]. It is further decreased to 44 emu/g for SPION-CCh/AA-Cur by the presence of the conjugate layer (AA-Cur). Nevertheless, it is sufficiently high for maintaining a strong magnetic response of the particles to external magnetic stimuli. 

In line with the size distribution of the particles probed with TEM, the size distribution of magnetic cores is also relatively narrow as evidenced by the temperature difference between the maximum of ZFC, T_m_ ~ 120 K and the coincidence temperature of the ZFC and FC curves. With a narrow size distribution and omitted interparticle interactions, it can be assumed that T_m_ ~ T_B_ × 1.06 [44]. Thus, the blocking temperature (T_B_) in both samples studied was estimated to be in the range of 90 ÷ 100 K. This is in line with the opened hysteresis loop probed at 80 K, i.e., below the blocking temperature.

### 3.3. Magnetic Heating

In an alternating magnetic field, the conversion of magnetic energy into heat is generally due to magnetization fluctuations within a nanoparticle. The heat could be generated during two different processes: relaxation and hysteresis loss. The physical mechanisms of relaxation leading to losses in iron oxides’ nanoparticles are based on Néelian and Brownian modes [45,46].

In magnetic hyperthermia (MHT), the temperature of the local environment of a tumor is raised by magnetic heating of nanoparticles, resulting in a change in the physiology of the cancer cells and, finally, leading to cell death [47,48]. Therefore, the obtained hybrid systems, SPION-CCh and SPION-CCh/AA-Cur, were tested as nanoheaters for potential applications in magnetic hyperthermia.

**Figure 8:**
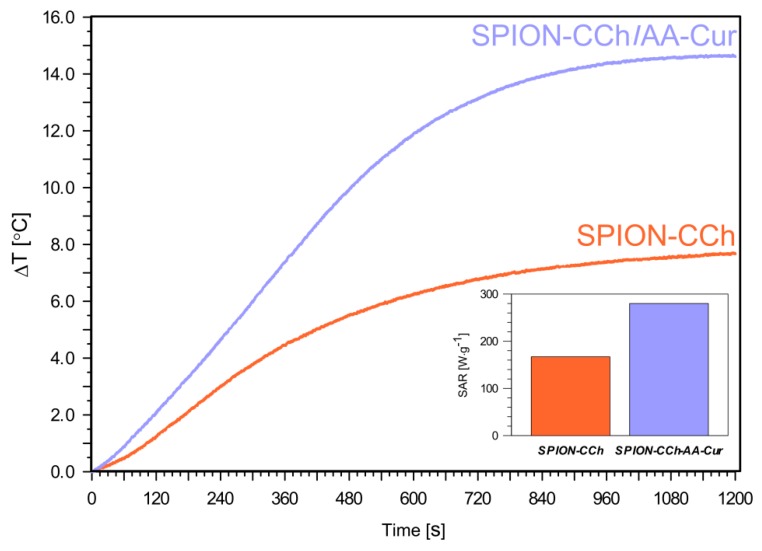
Temperature increase during 1200 s of magnetic heating of SPION-CCh and SPION-CCh-AA-Cur in water at c_Fe_ = 0.3 mg/mL.

The temperature variations of the suspensions of obtained systems (SPION-CCh and SPION-CCh-AA-Cur) at the same iron concentration (0.3 mg Fe/mL) were measured after their exposure to an oscillating magnetic field (23.77 kA/m, 360 kHz) (Figure 8). Both obtained systems showed good colloidal stability during the magnetic heating measurements (no sedimentation occurred during and after the measurements). The temperature increments (ΔT) for SPION-CCh and SPION-CCh/AA-Cur in water are 7.5 °C and 14.5 °C (c_Fe_ = 0.3 mg/mL), respectively. A plateau for both samples was reached after 1000 s of exposure to the magnetic field. The SPION-CCh/AA-Cur system achieved a temperature of 42–43 °C (the temperature of hyperthermia) even at low concentrations of iron.

The value of the specific absorption rate (SAR) was determined based on the observed increase in temperature. It was calculated using the following equation:

(1) $$\;SAR=C\cdot \frac{\Delta T}{\Delta t}\cdot \frac{1}{m_{mag}}\;$$

where: *C* is the heat capacity of the media (for water C = 4.189 J/g K), *T*—temperature (K), *t—*time (s), and *m_mag_*—mass of the iron in the sample. 

A SAR value of 280 ± 3 W⋅g^−1^ was obtained for SPION-CCh/AA-Cur at an iron concentration of 0.3 mg/ml (inset in Figure 8). The SAR value calculated for the SPION-CCh system, 168 ± 2 W⋅g^−1^, is much lower for the same iron concentration. This value, however, is higher than that reported for iron oxide nanoparticles with diameters between 8 and 11 nm [49] and for similar polysaccharide-coated SPION (including commercially available nanoparticles) or in similar conditions [50,51]. The obtained high SAR values were achieved likely due to a combination of a narrow distribution of the particles sizes, significant magnetic saturation, and very good colloidal stability [52]. The SAR values for our systems were slightly higher compared to the results obtained by other groups [53,54], possibly due to their ability to form small agglomerates within which microfriction necessary to generate the heat increases. Moreover, some research groups have indicated that nano-assemblies and chain-like structures can also lead to an increase in the heating efficiency of magnetic nanoparticles [55,56]. Another reason may be that when the nanoparticles are synthetized in the polymer matrix, a formation of chains with a uniaxial anisotropy without the presence of an AC field is possible [57]. This type of structure organization causes an enhancement of the effective anisotropy of the cores due to the unidirectional magnetization orientation as an effect of the dipolar coupling along the chain [58]. As a result, it can lead to the hysteretic losses, which can improve the energy dissipation process. We suppose that the SPION-CCh-AA-Cur system is more compact as a result of strong electrostatic interactions, thus, as the magnetic cores are closer, the probability of formation of chains with uniaxial anisotropy increases.

### 3.4. Fluorescence Measurements 

**Figure 9:**
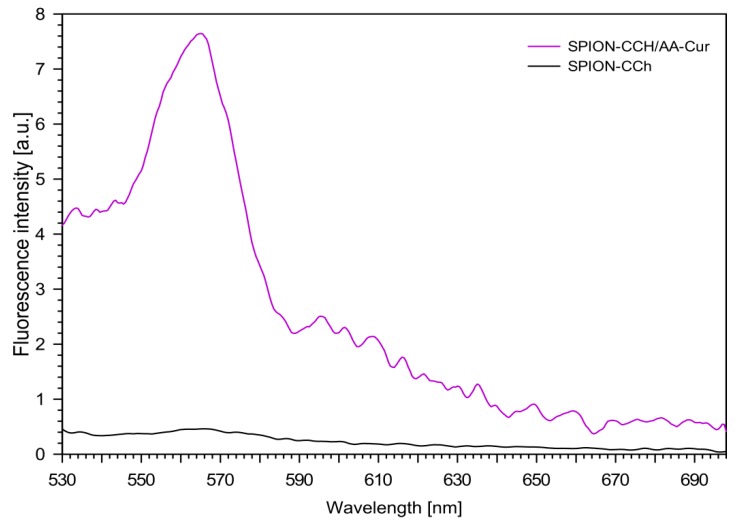
Fluorescence spectra of obtained systems (SPION-CCh, SPION-CCh/AA-Cur) in aqueous dispersions (c = 0.1 mM, λ_ex_ = 405 nm).

The fluorescence spectra of the obtained nanoparticulate systems (SPION-CCh and SPION-CCh/AA-Cur) were measured with excitation at 405 nm (maximum of curcumin absorption), and the results are shown in Figure 9. For the aqueous dispersion of the SPION-CCh system, no fluorescence was observed, as expected. For the SPION-CCh/AA-Cur suspension, a well visible fluorescence band, with maximum at 565 nm, was registered, confirming the presence of curcumin in the system. 

**Figure 10:**
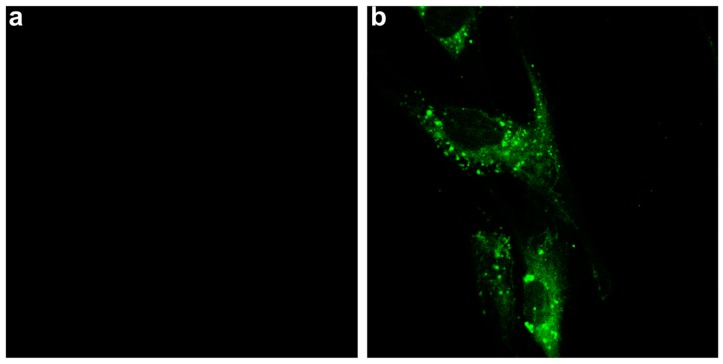
Confocal images of the cells (100×/1.4 Oil DIC) recorded before (**a**) and 8 h after treatment with (**b**) SPION-CCh/AA-Cur (0.3 mg/mL Fe). Images were obtained with excitation at 408 nm; emission was registered in the range of 480–540 nm.

The characteristic fluorescence of the SPION-CCh/AA-Cur system was used to study the cellular uptake and the location of the nanoparticles in fibroblast cells by confocal microscopy. Figure 10 shows the fluorescence image of fibroblasts before (a) and after (b) 8 h of incubation with SPION-CCh/AA-Cur. Bright green fluorescence observed exclusively inside cells confirms the effective uptake of the curcumin-containing system by fibroblasts. The observed uptake was fast compared to similar systems, e.g., only a low number of FITC-labelled SPION coated with PEG350 and PEG2000 were observed in fibroblasts after 24 h of incubation [59]. 

### 3.5. Biological Studies 

**Figure 11:**
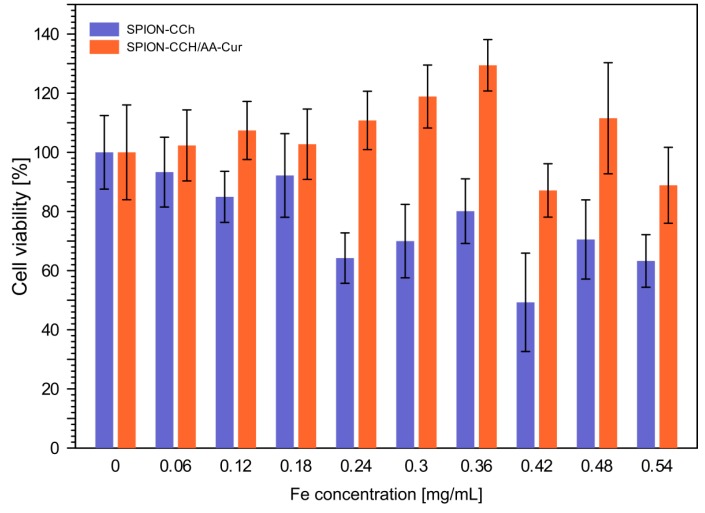
Results of the MTT assay conducted for NIH3T3 cells incubated for 24 h with SPION-CCh and SPION-CCh/AA-Cur at different concentrations of iron. Values are expressed as a percentage of the control, which was defined as 100%. The error bars represent mean and standard deviations of the experiments performed in triplicate (n = 3).

The obtained systems have potential biomedical applications; therefore, their cytotoxicity was also studied. The viability of murine fibroblast NIH3T3 cells treated with SPION-CCh and SPION-CCh/AA-Cur was determined. MTT test confirmed that, in the concentration used for hyperthermia (0.5 mg/mL), the SPION-CCh/AA-Cur system does not exhibit cytotoxicity. The results presented in Figure 11 indicate that there is a significant difference in the cytotoxicity between SPION-CCh and SPION-CCh/AA-Cur. The higher cytotoxicity of SPION-CCh is, with high probability, caused by the presence of cationic groups on the nanoparticles’ surface. It is known that cationic derivatives of chitosan (due to the presence of the quaternary ammonium groups) [60,61] may be cytotoxic to various types of cells. However, coating by conjugate AA-Cur considerably decreased this toxic effect. 

## 4. Conclusions

We have successfully decorated the surface of SPION with a bilayer polymeric coating consisting of polyions. The resulting SPION-CCh/AA-Cur system was colloidally stable and contained anti-cancer agent, curcumin, in the form of water soluble conjugate. TEM and SAXS analyses revealed the presence of agglomerates of elementary particles which, in turn, consisted of an iron oxide core and polymeric coating. The size of these elementary particles was estimated to be ca. 19 nm, while the size of the agglomerates was ca. 50 nm. To confirm the presence of both layers (CCh and AA-Cur), FTIR and XPS spectroscopies were used, complemented by TGA analysis. All three methods allowed us to confirm that both polymers were indeed present in the system studied. Fluorescence studies showed further that curcumin was indeed present in this system

The superparamagnetic properties of SPION-CCh/AA-Cur were confirmed by VSM, although it should be noted that their saturation magnetization decreased due to of the presence of the AA-Cur layer. Magnetic heating was also studied in order to verify the usefulness of SPION-CCh/AA-Cur in MHT. Our system showed good colloidal stability during the magnetic heating measurements. The temperature of 42–43 °C (the temperature of hyperthermia) was achieved for our system even at relatively low concentrations of iron. The high value of SAR (280 [W/g]) was measured for our system. Preliminary biological studies confirmed the effective uptake of SPION-CCh/AA-Cur and its significantly lower cytotoxicity toward fibroblasts compared to SPION-CCh.

Our studies have confirmed that the newly obtained SPION-CCh/AA-Cur nanoparticulate system is a promising magnetic hyperthermia agent. It also contains curcumin in the form of a water-soluble conjugate. By combining these two unique properties, our system shows high potential as an effective anti-cancer agent.

## Figures and Tables

**Table 1:** The hydrodynamic diameters and zeta potentials of SPION-CCh and SPION-CCh/AA-Cur.

Sample	d_max_ [nm]	PDI	ζ [mV]
SPION-CCh	108 ± 4	0.26	+34 ± 6
SPION-CCh-AA-Cur	58 ± 9	0.21	−35 ± 6
